# Wireless Signal Propagation Prediction Based on Computer Vision Sensing Technology for Forestry Security Monitoring

**DOI:** 10.3390/s21175688

**Published:** 2021-08-24

**Authors:** Jialuan He, Zirui Xing, Tianqi Xiang, Xin Zhang, Yinghai Zhou, Chuanyu Xi, Hai Lu

**Affiliations:** 1 School of Mechanical Electronic & Information Engineering, China University of Mining & Technology, Beijing 100083, China; hejialuan@163.com; 2 Beijing Aerocim Technology Co., Ltd., Beijing 102308, China; xingzirui1001@126.com; 3 School of Information and Communication Engineering, Beijing University of Posts and Telecommunications, Beijing 100876, China; xiangtianqi@bupt.edu.cn (T.X.); zhangxin@bupt.edu.cn (X.Z.); 4 China Academy of Engineer Physics, Institute of Computer Application, Mianyang 621054, China; zhouyinghai19@gscaep.ac.cn (Y.Z.); xicy@caep.cn (C.X.)

**Keywords:** CV sensing technology, wireless signal, forestry security monitoring, diffraction loss, shadow fading, convolutional neural network

## Abstract

In this paper, Computer Vision (CV) sensing technology based on Convolutional Neural Network (CNN) is introduced to process topographic maps for predicting wireless signal propagation models, which are applied in the field of forestry security monitoring. In this way, the terrain-related radio propagation characteristic including diffraction loss and shadow fading correlation distance can be predicted or extracted accurately and efficiently. Two data sets are generated for the two prediction tasks, respectively, and are used to train the CNN. To enhance the efficiency for the CNN to predict diffraction losses, multiple output values for different locations on the map are obtained in parallel by the CNN to greatly boost the calculation speed. The proposed scheme achieved a good performance in terms of prediction accuracy and efficiency. For the diffraction loss prediction task, 50% of the normalized prediction error was less than 0.518%, and 95% of the normalized prediction error was less than 8.238%. For the correlation distance extraction task, 50% of the normalized prediction error was less than 1.747%, and 95% of the normalized prediction error was less than 6.423%. Moreover, diffraction losses at 100 positions were predicted simultaneously in one run of CNN under the settings in this paper, for which the processing time of one map is about 6.28 ms, and the average processing time of one location point can be as low as 62.8 us. This paper shows that our proposed CV sensing technology is more efficient in processing geographic information in the target area. Combining a convolutional neural network to realize the close coupling of a prediction model and geographic information, it improves the efficiency and accuracy of prediction.

## 1. Introduction

Sensing technology plays an increasingly important role in many fields, especially in the field of public security. Among them, sensing technology is widely used in forestry safety monitoring systems. With the continuous development of afforestation, the area of forest land has been increasing year by year, and fire prevention has become the focus of forestry safety work. Forest fires have the characteristics of being sudden, random, and can cause huge losses in a short time. For this reason, video acquisition and coding are carried out at the monitoring points in important forestry areas by placing cameras, and the monitoring system server of the monitoring center is connected through wireless channels, while the video monitoring system software is used for centralized display and unified management. In the construction process of forestry security monitoring system, ensuring that the cameras are always in the signal coverage range of the central base station and other wireless communication facilities is the basis of guaranteeing the video transmission back. Therefore, it is necessary to quickly and accurately predict the transmission range of wireless signals before the base station and cameras are laid out. Furthermore, with each camera as the center, the intersection area of the wireless signal propagation range can provide support for the site selection of the base station. On the contrary, the location of the camera can be supported by the propagation range of the wireless signal centered on the base station where the location is determined.

Prediction or evaluation of wireless physical signal security propagation characteristics plays an important role in planning and deploying wireless networks [1], especially for scenarios such as hilly terrains [2,3], which require highly efficient modeling and calculation with acceptable accuracy. Traditional models are based on simplified classification of terrains, not reflecting the detailed distribution of terrain profiles or the objects on them, whose complexities are low at the cost of poor precision. More complicated modeling of electro-magnetic fields, e.g., the methods based on ray-tracing or ray-launching, however, bring high accuracy of prediction, but with prohibitive calculation complexity when the number of rays are huge [4,5]. Furthermore, when there are more and more wireless nodes networking and operating together, such as in a scenario of Internet of Things, the propagation conditions among those nodes would require the knowledge of channels from multi-point to multi-point. For instance, as ensemble classification theory [6] was applied to address some technological issues in Internet of Things networks [7], radio channel qualities related to massive users would be important for the system modeling and performance optimization, which poses even more challenges on improving the efficiency of predicting radio channels for a huge amount of wireless nodes.

With the fast development and application of Artificial Neural Network (ANN) in many technical areas, especially with deep learning [8], some research has also been conducted to predict wireless signal propagation [9,10,11,12], including ultra-high frequency bands over irregular terrains. It is known that ANN may have considerable advantage to improve the current propagation models. Drive tests would be carried out in cellular networks to obtain measurement results for calibrating propagation models and evaluating coverage performance, in which the collected data can be applied for training ANN models and even related prediction tasks. Compared with the traditional propagation prediction modelling, ANN generally shows better efficiency, especially with the aid of multi-thread computation capabilities of a Graphic Processing Unit (GPU), leading to highly efficient well-trained ANN models.

In the research related to radio channel modelling [13,14,15], typical ANNs can be trained for different mapping functions, and the inputs of the functions are generally a sequence of data samples varying over time or location, such as signal strengths values or path loss values from a driving test, with the outputs following a tendency predicted by the ANN. But in this process, the distribution of blocking obstacles, or irregular terrain patterns that have direct impact over propagation models, are not explicitly considered or modelled [16,17,18], therefore, the ANNs’ capabilities are mostly represented by extracting the varying trends of the train sets as well as matching the statistical properties between the train sets and the predicted results. Consequently, once the propagation scenarios change, e.g., the coverage area of interest is shifted from the driving test routes, or the mobility parameters are different from those in the train set, the prediction precision would degrade. In other words, the traditional application of ANN in propagation modelling does not directly process the terrain patterns or obstacles’ distribution, but just deal with an abstracted model from the limited training data with reduced adaptability for different and extensive scenarios, as the drive tests are not supposed to incorporate all the coverage area. Besides, the errors in the train sets caused by non-ideal measurement conditions, will also make the prediction less accurate. Even though there is research [1] on terrain-based propagation loss prediction with ANN, it still deals with one-dimensional terrain fluctuation, which did not consider the influence of non-radial profile terrain between transceivers, and still learns the trend of loss variation, unable to introduce a more accurate model.

To address the issues above with solutions different from typical methods of ANNs, this paper introduces the ANNs from Computer Vision (CV) that can process images or maps to detect and identify ground objects or terrain patterns, with reasonable modification and improvement, to design two basic CV-based propagation (parameters) prediction schemes. The proposed schemes seem preliminary, but the models underlying them are fundamental, which utilizes map data with terrain information as inputs, and process the propagation related factors represented by the map similarly to the way CV does in object detection or tracking. One proposed scheme maps these factors to a propagation/diffraction loss model, which could also be extended to a more generalized path loss prediction model beyond diffraction effect for irregular terrains with larger area. Another proposed scheme extracts the stochastic properties of shadow fading such as correlation distance, from topographic maps by CV.

The aim of the study is to establish a framework and method for CV-based terrain-related propagation model parameters prediction, including diffraction loss, shadow fading correlation distance, etc., as well as to verify its validation and efficiency.

There are some related works on applying CV in wireless communications [19,20,21,22,23], including feature extraction of signals’ time-frequency spectra, e.g., to generate a fast-fading channel model with the same stochastic properties as a real wireless channel by constructing images from the channel’s time-frequency responses with Generative Adversarial Networks [24]; or fast fading model establishment based on Simultaneous Localization And Mapping (SLAM) [25] in which CV is employed to detect objects and reconstruct the 3D spatial model to derive the multi-path properties. But these studies are not considering the large scale propagation models over complex terrains, which differ from the schemes of this paper. To be specific, the main contributions are as follows:

- Our proposed schemes directly process the maps with terrain information by Convolutional Neural Network (CNN) to obtain large-scale propagation/diffraction loss or shadow fading parameters. A framework including data set generation, network structure, training, and metrics evaluation has been constructed to research into the combination of CV and terrain-related radio propagation.
- A direct link is established by a CNN between topographic map to propagation/diffraction loss for a pair of transmitter and receiver on the map. Furthermore, the pathloss between a transmitter and multiple receivers can be predicted in one batch by taking advantage of the multiple parallel outputs defined for the CNN, which greatly enhances the computation efficiency and lays the foundation for extending the scheme to predict the pathloss from a transmitter to a coverage area in a very fast way.
- The quantitative relation is found between the terrain fluctuation pattern to correlation distance of shadow fading through a CNN model that can process a map of a coverage area, and the results would help configure radio access networks, e.g., to optimize handover performance.

These two schemes and use cases validate and demonstrate the great potential of CV in this research direction. Even though the paper launches the study from a quite basic propagation model in a limited coverage area, it can help establish a general CNN-based prediction model for further improvement via fine-tuning or transfer learning, as well as better understand the essence of the one-step prediction schemes and gain a deep insight about the most important factors involved, which deals with exploitation of the advantage of CNN in detection of 2-D and even 3-D patterns over a reasonable balance between prediction accuracy and computation efficiency.

ANN demonstrates great potential in predicting wireless physical signal security propagation characteristics also in other technical areas. Generally speaking, the employment of ANN for radio channel prediction is limited to learning, tracking, and predicting the variation of path loss or fading based on some drive test results or theoretical models, which does not directly process and exploit the terrain profile or ground object distributions that affect radio propagation. The complex terrain environment in forest areas has great influence on wireless signal propagation. The traditional forecasting methods are insufficient in predicting efficiency or coupling degree with terrain. Computer vision sensing technology is more efficient in processing the geographic information of the target area. Combined with the wireless signal coverage prediction based on CNN, the prediction model can be closely coupled with the geographic information, and the efficiency, accuracy, and adaptability of the prediction can be improved, which provides an important support for the construction of forestry safety monitoring system.

The remainder of the paper is organized as follows, Section 2 introduces the CNN-based diffraction loss prediction scheme, Section 3 describes the correlation distance extraction scheme for shadow fading based on CNN, Section 4 provides simulation results with analysis, Section 5 discusses some potential future works, and Section 6 concludes the paper.

## 2. Diffraction Loss Prediction Based on CNN

We get started from diffraction loss prediction by studying how to adopt CV sensing technology to participate in the establishment of a radio propagation model according to the terrain profile or blocking obstacles information shown in a map, and it is believed that the proposed scheme could be easily extended to scenarios with larger coverage areas and more complicated terrain properties as long as the data set is large and accurate enough. So it can provide more adaptive ability for the prediction of wireless signal propagation range.

Diffraction is an important and complex mechanism in electro-magnetic wave propagation, especially for signals with relatively longer wavelengths such as ultra-short waves, which allow the radio signal to transmit around the obstacles and form a signal coverage behind the obstacles. In Huygen’ theorem [26], diffraction is modelled by treating the points at the obstacles’ edge as new sources of wavefront that bring the waves to the shadow of the obstacles. In an irregular or hilly terrain, the line of sight path will be blocked by the mountains, thus, the propagation relies heavily on diffraction, and the losses are mainly caused by diffraction. It should be noted that the diffraction loss is related to signal wavelength, sizes of the obstacles, and their geometric distribution, as well as the electrical properties of the materials involved, which is often combined with reflections forming multi-path propagation. So the key to applying CV sensing technology to predict diffraction loss is, how to properly map those factors mentioned above in the 3D space to the overall loss value, especially in a single step, by necessary adaptation of traditional CNNs for processing topographic maps.

### 2.1. Prediction Method

**Figure 1:**
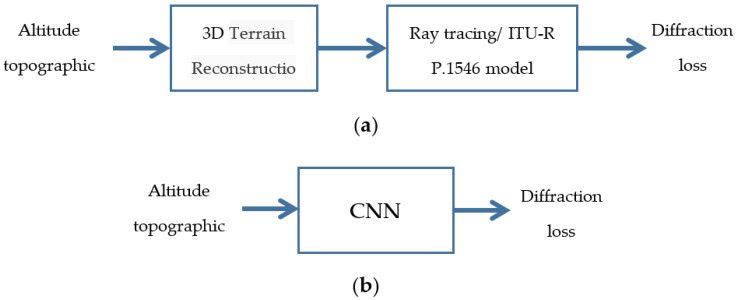
Loss prediction method based on altitude topographic map. (**a**) Model-based method; (**b**) CNN-based method.

CNN is proposed to be applied for diffraction loss prediction, and the well trained CNN will process a topographic map to generate the loss value between the transmitter and receiver marked on the map. When the altitude profile of an irregular terrain is used for path loss prediction, ray-tracing based method or ITU-R P.1546 model [27] requires some form of 3D reconstruction for the particular scenario before the loss value could be computed, which involves two steps of operation with considerable complexity for calculation of the output just between two positions, being not suitable for efficient predictions between one position to multiple positions or an area as in radio network planning process. Ray-tracing has higher accuracy, but it seems unable to identify and filter the main obstacles’ blockage effect with high efficiency to find the rays that make the most contribution for propagation, which makes it hard to reduce the complexity. But when CNN is adopted, it can exploit its advantage to extract the objects’ properties such as shapes and boundaries, which represent the terrain profiles directly affecting propagation loss, so we can train the CNN to extract these properties, then map them to a pathloss value, for which a complicated non-linear relation may function behind it. Besides the simple one-step operation of CNN from map to loss, it is proposed to use CNN to predict multiple loss values in parallel along the path from transmitter to receiver to further enhance the efficiency. Figure 1 presents these two methods. Though performing multiple predictions at one time would require a more complicated CNN structure, the CNN’s inherent feature that can support multiple output values would balance this negative effect, and with the aid of parallel computing power of GPU, the proposed scheme will significantly increase the prediction efficiency while keeping a good accuracy level.

### 2.2. Data Set Generation

**Figure 2:**
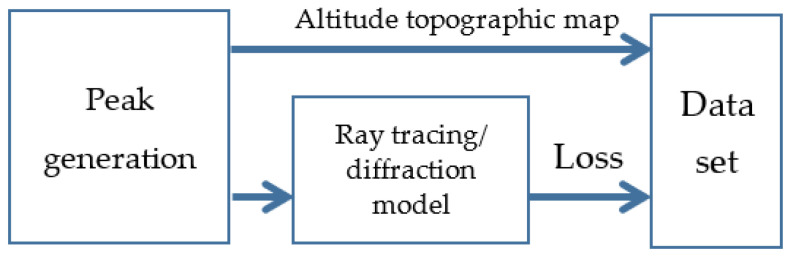
Data set generation.

Data set is generated according to Figure 2. First, a mountain peak with random altitude is generated, and its terrain profile information is recorded in an image, where each pixel with a certain grey level corresponds to the altitude value at that location. Then a traditional diffraction model is used for each picture to calculate the diffraction losses as the labels for CNN to process in the next step. It should be noted that, if field measurement results are available, they can also be employed as labels for training the CNN. This process will be repeated until the data set of maps is large enough. In this paper, 1000 maps are generated for training, 200 maps for testing, and 100 maps for validation. The data set with *n* = 1300 maps is represented by $D_{L}=\left\{\left\{M_{1},L_{1}\right\},\left\{M_{2},L_{2}\right\},\ldots ,\left\{M_{N},L_{N}\right\}\right\}$; For the *n*-th map, $M_{n}\in R^{X\times Y}$, $L_{n}\in R^{N_{L}}$ with X×Y altitude pixels and *N_L_* diffraction losses recorded.

**Figure 3:**
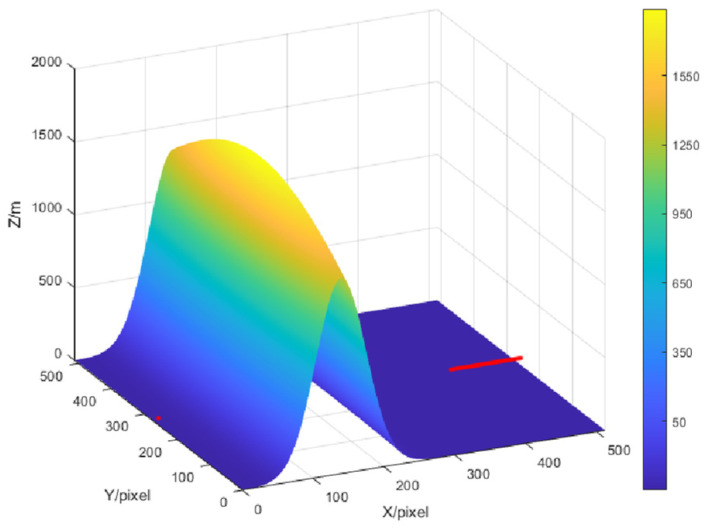
Generated topographic map diagram.

Figure 3 shows an example of the topographic map in the data set with 3D form, which is generated in a way specified as follows. A mountain is modeled in a map with size of *X* = 512 by *Y* = 512 pixels, and the peak amplitude is uniformly distributed in the range of (500 m, 2000 m); the peak is located in the center along the *y*-axis, but located in a random position along the *x*-axis. One pixel in the map represents 5 m. This mountain simulates the terrain of a typical mountain forest farm. The coordinate of the transmitter is at (0, 256, 10) in pixels. To obtain multiple diffraction loss values in the map in parallel, multiple labels will be calculated correspondingly, where the assumed multiple receivers are deployed along the line between position (413, 156, 10) and position (512, 156, 10), with a space of one pixel between the adjacent two of them, as indicated by the short red line in Figure 3, i.e., 100 labels will be needed for the CNN to output *N_L_* = 100 loss values simultaneously.

When the topographic maps are generated, a typical single-edge diffraction model is used to calculate multiple labels of the loss values. This paper proposes a generalized prediction framework using CV to focus on the validation, efficiency, and generalization ability of the proposed model. Therefore, this paper trains CNN network with a general diffraction loss model, so as to predict the diffraction loss under most topographic conditions. When this model is applied to a practical system with unique terrain-related wireless propagation characteristics, as consequences of unique vegetation and soil, field collected data can be used to learn new terrain-related features through fine-tuning or transfer learning on the basis of the proposed generalized CNN.

**Figure 4:**
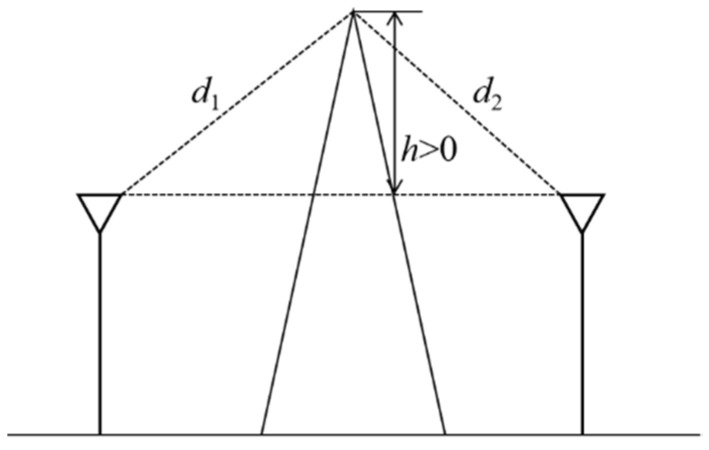
Parameters used in diffraction loss equation.

In this paper, the diffraction loss model is determined by a single parameter *v* that combines all the geometric parameters involved, and calculated as follows,

(1) $$J\left(v\right)=-20\log\left(J^{\prime }\left(v\right)\right)\left[dB\right]$$

where

(2) $$J^{\prime }(v)=\frac{\sqrt{{\left[1-C(v)-S(v)\right]}^{2}+{\left[C(v)-S(v)\right]}^{2}}}{2}$$

(3) $$S(v)=\int_{0}^{v}\sin(\frac{\pi s^{2}}{2})ds$$

(4) $$C(v)=\int_{0}^{v}\cos(\frac{\pi s^{2}}{2})ds$$

(5) $$v=\lambda \sqrt{2[\left(d_{1}+d_{2}\right)/d_{1}d_{2}]}$$

where *λ = c/f* is wavelength with *c* as light of speed and *f* as the radio frequency; geometrical parameters *h*, *d*_1_, *d*_2_ are illustrated as in Figure 4. *h* represents the height above the connection of transmitter and receiver and *d*_1_ and *d*_2_ are the distances between transmitter and the knife edge and receiver and the knife edge, respectively. A single parameter *v* combining all the geometric parameters is first derived by Equation (5); then Equations (3) and (4) give the sine term and cosine term of Fresnel integral respectively; the linear value of diffraction loss is calculated by Equation (2) with the two terms of Fresnel integral; finally the dB value of diffraction loss is derived by Equation (1).

### 2.3. CNN Structure and Performance Metrics

**Figure 5:**
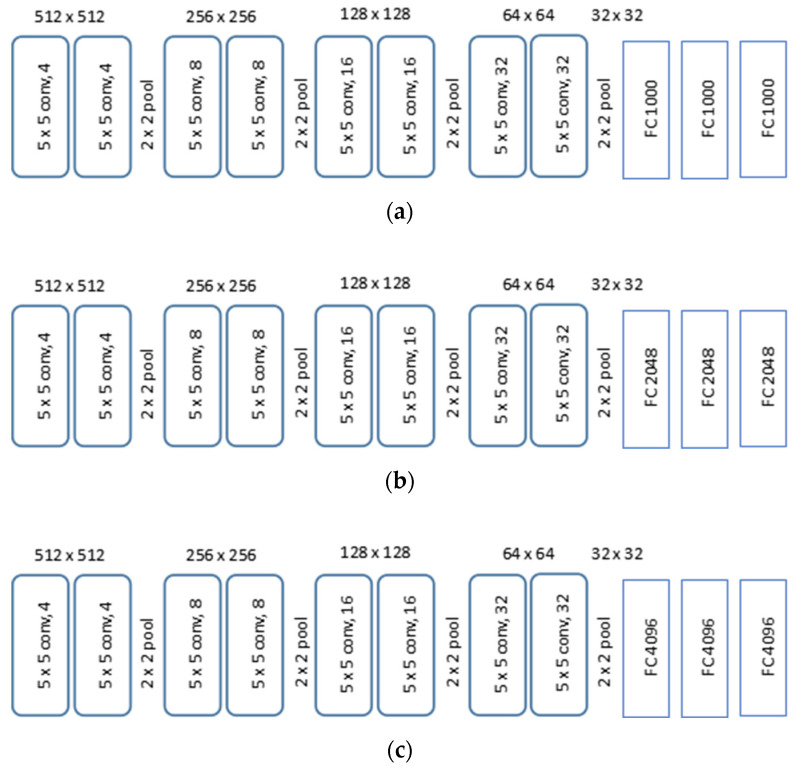
Structure of diffraction loss prediction CNN. (**a**) Net A; (**b**) Net B; (**c**) Net C.

A CNN structure similar to VGG (Visual Geometry Group) is adopted as shown in Figure 5. A typical VGG structure consists of several groups of convolutional layers followed with a pooling layer as well as several fully connected layers at the end of the network. The convolutional-and-pooling layer groups are capable of 2D image information extraction such as object edges detection and terrain parameter extraction; the fully connected layers are able to construct complex mapping functions, for instance, from terrain parameters to terrain-related diffraction loss. The ReLu activation function is adopted after each convolutional layer and fully connected layer, which is also known as ramp function. Considering that the geometric parameter extraction of the terrain profile would not be a challenging task due to the simple layout of the single edge obstacle, we employ a network structure different from typical VGG, i.e., a 5 × 5 convolution core is used in convolutional layer with relatively less channels. As this prediction task poses a high precision requirement for the loss model, several neural networks with different numbers of neurons are investigated in the full connection layer. The purpose of designing these networks is to verify whether more neurons would have the ability to better fit the mapping from geometric parameters to diffraction loss, so as to guide further network design in similar tasks using ANN. The input is single-channel topographic maps in the size of 512 × 512 pixels. In Figure 5, “5 × 5 conv, 4” means a convolutional layer with 5 × 5 convolution core and 4 channels; “2 × 2 pool” represents a pooling layer with a 2 × 2 max pooling core; “FC 1000” stands for a fully connected layer with 1000 neurons. Moreover, after each pooling layer, the length and width of the map are halved, for instance, from “512 × 512” to “256 × 256” after the first pooling layer. The output is a series of predicted diffraction losses, with the total number *N_L_* = 100. The loss function is defined as mean square error (MSE) between the predicted loss value and the corresponding label value, both in dB, expressed by

(6) $$\mathrm{Loss}=\frac{1}{N_{L}}\sum_{i=1}^{N_{L}}{\left({\hat{l}}_{i}-l_{i}\right)}^{2}$$

where *i* is the index of a predicted loss for a receiver position among all the *N_L_* locations, $l_{i}$ is the label’s value, and ${\hat{l}}_{i}$ is the predicted loss value.

Two performance metrics are used to evaluate the accuracy of the neural network after training: the distribution of absolute errors and the distribution of normalized errors obtained by test data set, respectively expressed as:

(7) $$\mathrm{absolute}\;\mathrm{error}\left(\mathrm{dB}\right)=\left|{\hat{l}}_{i}-l_{i}\right|$$

(8) $$\mathrm{normalized}\;\mathrm{error}\left(\%\right)=\frac{\left|{\hat{l}}_{i}-l_{i}\right|}{l_{i}}$$

## 3. Correlation Distance Prediction Based on CNN for Shadow Fading

### 3.1. Prediction Method

The correlation distance of shadow fading is important for coverage analysis or optimizing of forest district wireless network structure. The prediction method about it is similar to that for diffraction loss, which is carried out by detecting and extracting the fluctuation properties of the terrain profile in a map. For instance, when a VGG network is used to process the topographic maps with grey-scale, some existing channel model such as ITU-R P.1546 or other deterministic models can be used as the reference to produce shadow fading values as labels, or field test results can be adopted as labels, to train the neural network for obtaining shadow fading parameters, i.e., correlation distance in this paper. However, if our main purpose is to validate the proposed CV sensing technology solution from a theoretical perspective and to gain some insight, we can make certain simplifications on the generation of the data set, in other words, shadow fading random variables will be generated for each pixel on the data set maps that follow certain statistical property, as these values are corresponding directly to the correlation distance of shadow fading caused by the fluctuating terrain profile or the objects on the ground. Accordingly, the maps of shadow fading will be used as the input to CNN, and the correlation distance of the shadow fading will serve as the output predicted by the CNN in just one step, as similar to Section 2.

### 3.2. Data Set Generation

The basic procedure here is similar to the one in Section 2.2, which generates a large number of maps with pixels being random shadow fading values. The correlation distance for the shadow fading values in one map is assumed to be fixed, but could be different from map to map. Then, the maps will be used as train set, test set, and validation set. The data set also includes 1000 maps for training, 200 for testing, and 100 for validation (*n* = 1300), represented by $D_{SF}=\left\{\left\{SF_{corr,1},d_{1}\right\},\left\{SF_{corr,2},d_{2}\right\},\ldots ,\left\{SF_{corr,N},d_{N}\right\}\right\}$; For the *n*-th map, $SF_{corr,n}\in R^{X^{\prime }\times Y^{\prime }}$, $d_{n}\in R$ with $X^{\prime }\times Y^{\prime }$ shadow fading pixels and a correlation distance recorded. To distinguish the coordinate symbols from Section 2, we use *X*′, *Y*′, *x*’, and *y*′ in this section.

The correlated shadow fading values are obtained according to [28]. An independent lognormally distributed random variable $SF_{uncorr,n,x^{\prime },y^{\prime }}\sim N\left(0,\sigma^{2}\right)$ is first generated for each grid in the map. In order to introduce correlation, the generated uncorrelated shadow fading maps are processed as follows:

(9) $$S_{Z}\left(u,v\right)={\left|H\left(u,v\right)\right|}^{2}S_{W}\left(u,v\right)$$

where

(10) $$H\left(u,v\right)=F_{2D}\left(SF_{uncorr}\right)$$

(11) $$S_{W}\left(u,v\right)=F_{2D}\left(R\right)$$

where $F_{2D}\left(•\right)$ is 2D discrete Fourier transformation, calculated as:

(12) $$F\left(u,v\right)=F_{2D}\left(f\left(x^{\prime },y^{\prime }\right)\right)=\sum_{x^{\prime }=0}^{X^{\prime }}\sum_{y^{\prime }=0}^{Y^{\prime }}f\left(x^{\prime },y^{\prime }\right)e^{-j2\pi \left(\frac{ux^{\prime }}{X^{\prime }},\frac{vy^{\prime }}{Y^{\prime }}\right)}$$

H(u,v) is the 2D discrete Fourier transformation of *X_k_*; $S_{W}\left(u,v\right)$ is the 2D Fourier transformation of a 2D correlation function, represented as below:

(13) $$R_{x^{\prime },y^{\prime }}=e^{-\frac{{\left\|\left(x^{\prime },y^{\prime }\right)\right\|}_{2}}{L}}$$

where *L* denotes the correlation distance of shadow fading and ${\left\|•\right\|}_{2}$ is the L2-norm operator. Finally, the correlated shadow fading map will be computed by 2D inverse discrete Fourier transformation of $S_{Z}\left(u,v\right)$:

(14) $$SF_{corr,n,x^{\prime },y^{\prime }}=F_{2D}^{-1}\left(S_{Z}\right)$$

and the 2D inverse discrete Fourier transformation is as follows:

(15) $$f\left(x^{\prime },y^{\prime }\right)=F_{2D}^{-1}\left(F\left(u,v\right)\right)=\frac{1}{X^{\prime }Y^{\prime }}\sum_{u=0}^{X^{\prime }}\sum_{v=0}^{Y^{\prime }}F\left(u,v\right)e^{j2\pi \left(\frac{ux^{\prime }}{X^{\prime }},\frac{vy^{\prime }}{Y^{\prime }}\right)}$$

**Figure 6:**
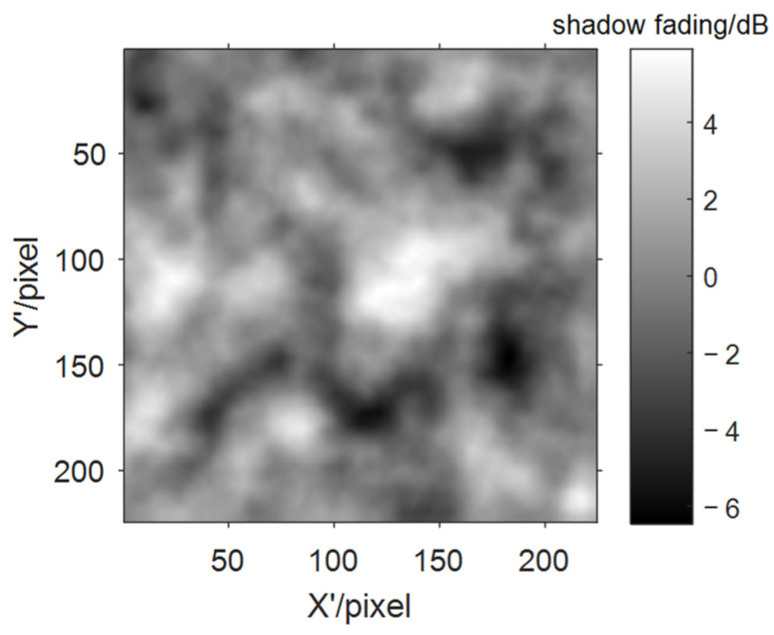
An example for shadow fading map.

In order to train the neural network to extract the correlation distance of shadow fading, we assume the correlation distance follows a uniform distribution in a certain range for the whole data set, and each map in the set has a unique correlation distance, but shares the same standard deviation for simplicity. An example of the shadow fading map is shown as in Figure 6.

### 3.3. CNN Structure and Performance Metrics

**Figure 7:**
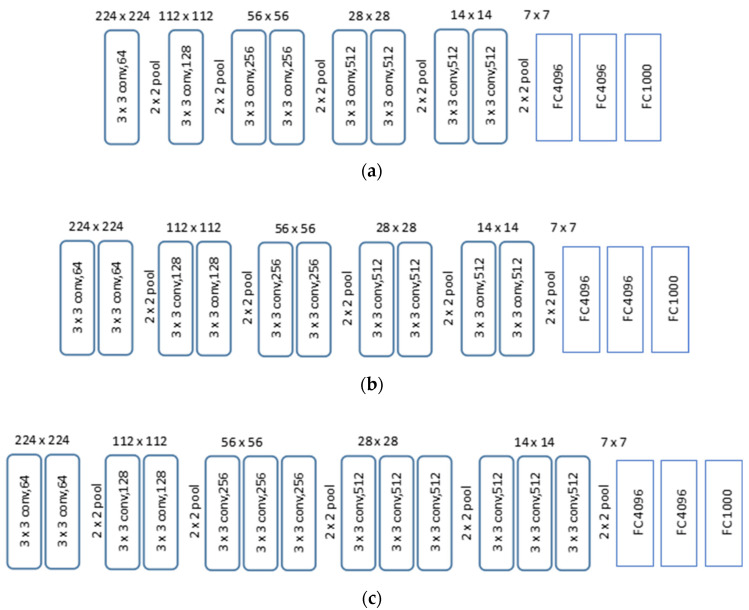
Structure of correlation distance extraction CNN. (**a**) Net D; (**b**) Net E; (**c**) Net F.

Figure 7 displays the VGG network structures under consideration, where net D, net E, and net F are based on VGG11, VGG13, and VGG 16 [29,30], respectively. The input map has 224 × 224 ($X^{\prime }\times Y^{\prime }$) pixels of shadow fading values, which will lead to a corresponding correlation distance as output. Different from the diffraction loss prediction task, the main challenge of correlation distance extraction is image pattern recognition rather than complex mapping fitting. Therefore, VGG11, VGG13, and VGG16 with different convolution layer depths are used to verify the impact of convolution layer depth on the performance of correlation distance pattern recognition. We also expect that this research will provide reference and guidance for further similar research.

Similar to Section 2.3, the MSE loss function is adopted as the criteria for training CNN. The distribution of absolute and normalized errors in test data set are used to evaluate the accuracy performance, respectively calculated as:

(16) $$\mathrm{absolute}\;\mathrm{error}=\left|\hat{d}-d\right|$$

(17) $$\mathrm{normalized}\;\mathrm{error}\left(\%\right)=\frac{\left|\hat{d}-d\right|}{d}$$

## 4. Simulation Results

The training and evaluation processes for the CNNs are based on the data sets generated as described in Section 2.2 and Section 3.2, respectively. For each task, in total, 1000 maps are generated as train set, 200 maps for test set, and 100 maps for validation set. Back propagation method [31] is adopted for CNN training in this paper, and the main hyperparameters for CNN training are shown in Table 1. The adopted training hyperparameters are determined after artificial attempts to ensure that the CNNs for the two tasks would have good performance after training. The second task uses a larger batch size (the number of pictures fed to the network during a parameter update process in back-propagation method) to learn general pattern characteristics of the images, while the first task uses a smaller batch size to fit the mapping from terrain to diffraction loss more accurately. The adoption of exponential decay learning rate can dynamically reduce the learning rate in the training process, so as to reduce the risk of over fitting and further improve the network performance. ReLu activation function and Adam optimizer are used to improve the training efficiency. Under the assumptions of the above training parameters, the two types of tasks converge at 200 and 100 epochs (the whole training data set is fed to the network once in a training epoch), respectively. The radio signal frequency is set to 600 MHz, which is capable of wide coverage in 5G frequency bands. The data sets are generated with Matlab; CNN is established, trained, and tested using Python with Tensorflow framework.

### 4.1. Results of Diffraction Loss Prediction

**Figure 8:**
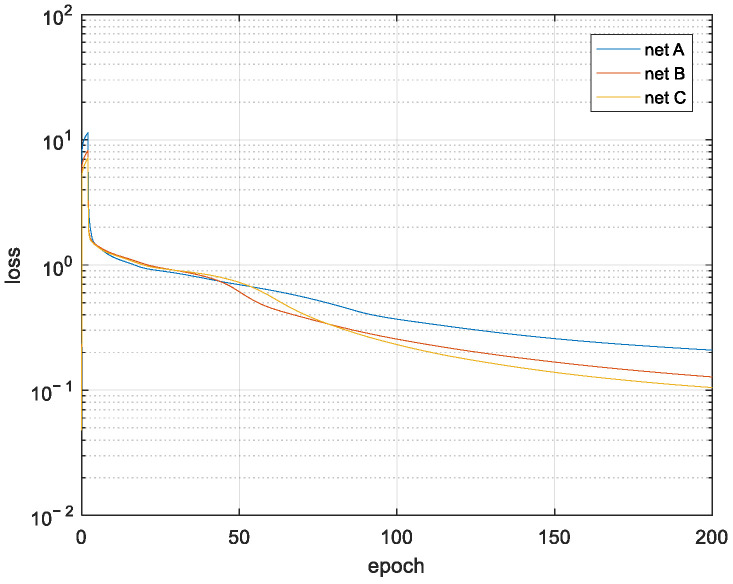
Training loss of diffraction loss prediction.

**Figure 9:**
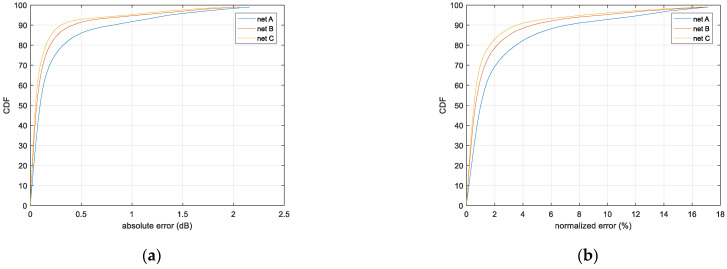
Error distribution of diffraction loss prediction. (**a**) Absolute error; (**b**) Normalized error.

Figure 8 gives the loss variations during the training iterations. Figure 9 compares the errors’ distributions for the three types of CNN structures after training, presented by cumulative distribution function (CDF) curves, and their performances are summarized in Table 2. It can be seen that, with the increasing number of neurons in the networks from net A to net C, the prediction accuracy is enhanced significantly, with a slightly prolonged processing time. Net C has the best error performance, while net A has the shortest processing time. The results in Table 2 were tested on the hardware platform of GTX1080Ti, with the powerful parallel processing capability of GPU and large memory. Even the computation workload of net B can be as twice as that of net A, but their processing times are almost the same. So the appropriate CNN structure should be carefully selected according to the specific error requirement of a pathloss prediction task as well as the computation power available.

To highlight the advantage of the proposed method, we also did some experiments about predicting just one loss value at a time with other conditions unchanged, which means that the CNN is simplified to output just one value at a time. In this case, the normalized error for 95% (percentile) samples is below 4%, and the processing time ranges from 6.15 to 6.3 ms. It can be observed that, the processing time per image or per map for the CNN with *n* parallel outputs is almost the same as that for the CNN with only one output, which can be translated to a nearly *n* (=100) times of efficiency enhancement for obtaining each pathloss value on one pixel. Meanwhile, the gain in computation efficiency comes at some cost of reduced accuracy, but it seems to be a reasonable and acceptable tradeoff, for example, regarding net C, its normalized error for 95% samples is below 8.238%, just a little higher than the error around 4% for the CNN with one output.

### 4.2. Results of Shadow Fading Correlation Distance Extraction

**Figure 10:**
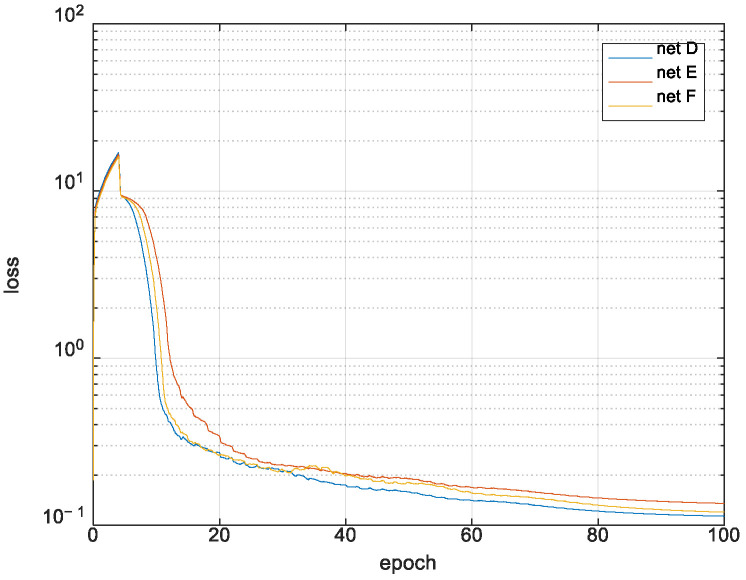
Training loss of correlation distance extraction.

**Figure 11:**
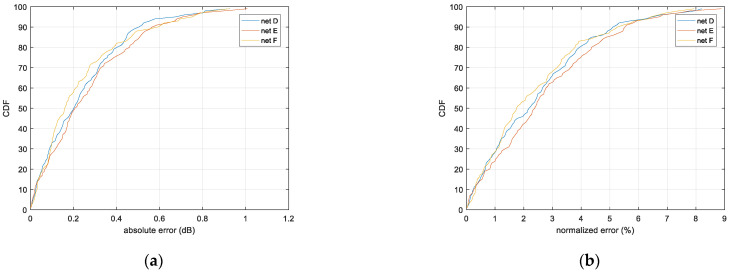
Error distribution of correlation distance extraction. (**a**) Absolute error; (**b**) Normalized error.

To generate the data set of shadow fading maps, the standard deviation is fixed at 2dB, and correlation distances are uniformly distributed in the range (5, 15) in the pixels. The size of the map is 224 × 224 pixels, and one pixel can correspond to different distances in the real world with varying scaling levels. The loss variation during the training process is shown in Figure 10, and the error performances are compared in Figure 11.

It can be seen from the results that, for extracting the correlation distance from shadow fading maps, the neural networks differ in convergence speed during training and in prediction error. Table 3 summarizes the error performances. For all the three networks, the 95% of samples have an error lower than 7%, and net F (VGG16) has the lowest median error; yet for other percentile values, net D indicates better performance. This is because Net F is able to learn more complex characteristics at the cost of losing universality, which might be attributed to the better fitting accuracy from improved depth of convolution layer; on the contrary, Net D is more capable from the perspective of universality. From this perspective, further study may be needed to optimize the CNN structure.

The correlation distance is deeply connected with the image patterns of the shadow fading map, which enables the CV sensing technology to take its advantage in object detection. But compared with the normalized error results of diffraction loss prediction, correlation distance prediction has a generally wider error distribution, which could also be explained as that, object detection would be more difficult on a random shadow fading map than on a topographic map with a single-edge-shaped mountain.

According to the discussion above, a preliminary observation could be made: for a terrain profile with significant 2D or 3D shape features, or for a terrain with less random properties, CV sensing technology would have more advantage to predict propagation loss-related parameters thanks to its inherent capability of object detection. For a real map that contains terrain or building information, though it looks ‘random’, the natural ground objects like rivers, mountains or valleys, etc., have some 2D or 3D features to be recognized or detected by CNN, not to mention the man-made buildings and roads with unique visual features. So we believe that the proposed basic scheme of using CV sensing technology to large and even small scale radio channel modeling would have great potential to predict the propagation range of wireless signals in more complex scenarios or larger geographical areas. Additionally, it has great significance to support the construction of a large area forest safety monitoring system.

## 5. Future Work

Based on the proposed modelling method and schemes, more research could be carried out in the future.

First, the proposed schemes in this paper can be extended to a more complicated scenario with longer distance between transmitter and receiver, e.g., for a propagation path with multiple mountains. We think that the prediction performance by CV sensing technology is mainly affected by the size of the data set and the accuracy of the labels. There would be two types of solutions to accomplish this. One is to introduce a more complicated convolution layer design to directly process maps of a larger area, with some pre-process of filtering to reduce the randomness of the large scale maps. The other is to divide the longer propagation path into several smaller segments, then let the less complicated CV sensing network to process these segments before the pathloss values in each segment could be accumulated to the overall loss value. For these two types of solutions, the former is expected to have lower error, but higher complexity, while the latter may have lower complexity but higher error, and how to optimize them or to search for other solutions needs further investigation, especially with the support of the field test results.

Second, the CV sensing network structures should be studied for their applications in a more practical and more complicated propagation scenarios. The neural networks in this paper can only process scenarios with fixed pre-configured parameters such as frequency, transmitter-receiver distance, and locations in grey scale maps, and are not able to be flexibly adjusted for varying parameter settings, or to exploit the multi-dimension information from colorful images. For example the current scheme may adopt a three channel image input with different colors to model the impact of air refraction or absorption. To flexibly support more diversified evaluation requirements, more inputs can be added to the neural networks, e.g., some inputs can be introduced at the beginning part of the fully connected layer to control the classification of operation for different frequency bands, or new inputs can be added for controlling map scales and modelling normalized locations for transmitters or receivers, etc. The data sets should also be extended correspondingly. All the potential issues involved would bring about more research directions in the future. The physical security propagation prediction method of wireless signal will also have stronger adaptability and accuracy in these research processes.

## 6. Conclusions

It is proposed in this paper that CNN structures can be applied in a novel way in CV sensing technology to process maps with terrain profiles or shadow fading information, so that the related pathloss model or statistical properties of shadow fading can be obtained directly, which can be used in a forest safety monitoring system. First, CNNs with multiple outputs are used to predict a batch of diffraction losses at multiple locations in a map with very high efficiency. The data set is generated in a form of maps containing random edge-shaped mountains, labelled by diffraction losses, calculated based on a typical mathematic diffraction model. Then, the CNNs are trained and tested to predict diffraction losses, with their structures being studied in terms of accuracy and efficiency. It is also discussed how to extend this type of CNNs to operate in more complicated propagation scenarios with longer distances. Second, CNNs are also adopted to extract shadow fading parameters such as correlation distances. Shadow fading maps are generated with fixed standard deviation and randomly configured correlation distances to create the data set. Then, the structures and performances of the trained CNNs are analyzed. Finally, through extensive comparisons and analysis, certain basic principles or potential guidelines are discussed for designing and applying CV sensing technology based on CNNs for a wireless physical signal security propagation model, followed by some related future works being provided. At the same time, this paper provides a theoretical support for the forestry security monitoring technology of wireless signal propagation prediction.

## Figures and Tables

**Table 1:** CNN hyperparameters.

Hyperparameters	Net A, B and C	Net D, E and F
batch size	1	16
initial learning rate	0.00001	0.0001
epoch	200	100
learning rate decay	exponential decay	
optimizer	Adam	
activation	ReLu	

**Table 2:** CNN performance of diffraction loss prediction.

		Net A	Net B	Net C
absolute error (dB)	50%	0.095	0.061	0.050
	90%	0.816	0.418	0.300
	95%	1.370	1.074	0.953
normalized error (%)	50%	1.006	0.632	0.518
	90%	7.120	4.655	3.577
	95%	12.434	9.634	8.238
processing time (ms)	per image	6.28	6.41	6.65
	per point	0.0628	0.0641	0.0665

**Table 3:** CNN performance of correlation distance extraction.

		Net D	Net E	Net F
absolute error (dB)	50%	0.210	0.207	0.159
	90%	0.503	0.565	0.591
	95%	0.673	0.708	0.752
normalized error (%)	50%	2.181	2.377	1.747
	90%	5.103	5.521	5.267
	95%	6.472	6.716	6.423

## Data Availability

Data sharing is not applicable to this article.
